# Real-world effectiveness of antiretroviral backbone regimens on viral suppression in pediatric and adolescent HIV care: a six-year cohort study from Uganda

**DOI:** 10.1016/j.eclinm.2026.103828

**Published:** 2026-03-12

**Authors:** Collins Ankunda, Brendah Kyomuhangi, Jude Emunyu, Sharon Namasambi, Conrad Sserunjogi, Jane Nakawesi

**Affiliations:** a Department of Global Public Health, Karolinska Institute, Stockholm, Sweden; b Department of Pharmacology and Therapeutics, Makerere University, Kampala, Uganda; c Mildmay Research Centre Uganda, Entebbe Rd, Naziba Hill, Kampala, Uganda; d Entebbe Regional Referral Hospital, Entebbe, Uganda; e Makerere University Walter Reed Program, Kampala, Uganda; f Uganda Public Health Fellowship Program, Uganda National Institute of Public Health, Kampala, Uganda; g Baylor College of Medicine Children's Foundation, Kampala, Uganda

**Keywords:** Pediatric and adolescent HIV, Antiretroviral therapy, Viral suppression, NRTI backbone, Real-world effectiveness, Uganda

## Abstract

**Background:**

Optimizing nucleoside reverse transcriptase inhibitor (NRTI) backbones is vital for pediatric and adolescent HIV treatment, yet real-world comparative evidence is limited. This study assessed backbone regimen effectiveness and factors influencing viral suppression (VLS) in Uganda.

**Methods:**

We conducted a retrospective cohort of 1033 children and adolescents (≤19 years) receiving HIV care in Uganda (2018–2023). Participants received Tenofovir Disoproxil Fumarate (TDF, 57.3%), Abacavir (ABC, 32.7%), or zidovudine (AZT, 10.0%). Viral load (VL) suppression was classified as suppressed (≤200 copies/mL), low-level viremia (201–999 copies/mL), and high-level viremia (≥1000 copies/mL). Outcomes were compared by χ^2^ tests, and mixed-effects logistic regression identified factors associated with non-suppression over time.

**Findings:**

Overall VLS was 83.5%. VLS was highest among TDF recipients (84.8%), followed by ABC (82.8%), and lowest with AZT (78.6%; p = 0.313). AZT recipients had the highest high-level viremia (19.4%) and greater odds of non-suppression compared with ABC (adjusted odds ratio [aOR] 2.02, 95% CI 1.20–3.40; p = 0.008). TDF was associated with lower odds of non-suppression in bivariable analysis (OR 0.71, 95% CI 0.53–0.95; p = 0.023 but was not significant after adjustment (p = 0.748). Increasing age reduced the odds of non-suppression by 8% per year (aOR 0.92, 95% CI 0.86–0.98; p = 0.006), while male sex increased odds of non-suppression (aOR 1.36, 95% CI 1.03–1.81; p = 0.033). Later VL testing time points were linked to improved suppression (aOR 0.95, 95% CI 0.91–1.00; p = 0.037).

**Interpretation:**

Overall VLS exceeded 80% but fell short of the UNAIDS 95% target. TDF-backbones had the highest success, AZT the lowest. Younger age and male sex predicted poorer outcomes. These results highlight the need for optimized regimens, targeted adherence support, and strengthened programs to improve pediatric and adolescent HIV care.

**Funding:**

10.13039/501100000272National Institute for Health and Care Research (NIHR), United Kingdom through the Royal Society for Tropical Medicine and Hygiene Early Career Grants.

**Research in context**

**Evidence before this study**

We searched PubMed, Embase, Web of Science, and Google Scholar from January 2000 to January 2026 using terms including “antiretroviral therapy”, “backbone regimen”, “children”, “adolescents”, “tenofovir”, “abacavir”, “zidovudine”, and “viral load suppression”, without language restrictions. Reference lists of key articles and World Health Organization (WHO) and national HIV guidelines were also screened. Most studies were cross-sectional or short-term clinical trials, with few longitudinal analyses of real-world cohorts. Evidence indicated tenofovir-based regimens generally achieved better viral suppression than zidovudine or abacavir in adults and older adolescents, while younger children showed more variable outcomes due to formulation and adherence challenges. Although pooled analyses report global viral suppression estimates in children and adolescents, no meta-analysis has directly compared tenofovir disoproxil fumarate (TDF), abacavir (ABC), or zidovudine (AZT) performance in this population.

**Added value of this study**

This study provides one of the largest and longest real-world cohort analyses of backbone antiretroviral therapy in children and adolescents in sub-Saharan Africa. Following over 1000 participants across six years, we demonstrate important differences in viral suppression outcomes by regimen. Tenofovir backbones consistently achieved the highest suppression, abacavir regimens performed moderately, and zidovudine regimens performed poorest, with higher rates of non-suppression even after adjusting for age, sex, and clinical factors. We also identify younger age and male sex as predictors of poorer outcomes, and show evidence of temporal improvement in suppression between 2018 and 2023, suggesting programmatic gains. These findings expand the evidence base by providing long-term, programmatic data relevant to real-world HIV care, beyond the controlled settings of clinical trials.

**Implications of all the available evidence**

Taken together with existing evidence, our findings reinforce the global shift away from zidovudine as a preferred backbone, highlight the continued importance of optimizing nucleoside reverse transcriptase inhibitor selection even in the dolutegravir era, and emphazise the need for targeted support for younger children and male adolescents. Policy makers and program implementers in resource-limited settings should prioritize tenofovir-based regimens where feasible, ensure availability of child-friendly formulations, and design age- and sex-sensitive adherence support interventions. Future research should focus on prospective evaluation of backbone choices in the context of dolutegravir roll-out, incorporating adherence metrics, resistance testing, and long-term clinical outcomes to guide sustained improvements in pediatric and adolescent HIV care.

## Introduction

Globally, an estimated over 1.5 million children and adolescents are living with HIV, with the vast majority residing in sub-Saharan Africa.^1^ Despite remarkable advances in antiretroviral therapy (ART) scale-up and the adoption of potent first-line regimens, viral load (VL) suppression in this age group remains suboptimal compared to adults.^1^ Achieving and maintaining VL suppression is critical for reducing HIV-related morbidity and mortality, preventing onward transmission, and improving long-term health outcomes. However, children and adolescents face unique challenges including perinatal infection, delayed diagnosis, complex dosing needs, limited pediatric drug formulations, and psychosocial factors such as dependence on caregivers for adherence.^2^^,^^3^

Backbone antiretroviral options, typically composed of nucleoside reverse transcriptase inhibitors (NRTIs), remain central to constructing effective ART regimens.^4^^,^^5^ The World Health Organization recommends tenofovir disoproxil fumarate (TDF), abacavir (ABC), or zidovudine (AZT) as backbone components, selected based on age, weight, prior drug exposure, and comorbidities.^6^, ^7^, ^8^ Although ABC and TDF are once-daily, TDF is preferred for potency, while ABC and AZT remain common in resource-limited pediatric care settings.^6^ Evidence comparing long-term VL outcomes across these backbones in real-world pediatric and adolescent cohorts remains limited.^9^ Most available studies are cross-sectional or short-term clinical trials, which may not capture the dynamic interplay between regimen choice, growth and development, caregiver support, and adherence challenges over time.^10^

In Uganda, where an estimated 71,000 children under 15 years are living with HIV, national guidelines have progressively aligned with WHO recommendations, promoting earlier initiation of ART and routine VL monitoring.^11^ Nonetheless, programmatic data indicate that adolescents and younger children lag behind adults in achieving sustained VL suppression, emphasizing the need to evaluate factors driving these disparities.^12^, ^13^, ^14^ Understanding the performance of different ART backbones in real world clinic setting is crucial for guiding regimen selection, improving treatment outcomes, and informing national and global policies, particularly in the current era where Dolutegravir (DTG) is the preferred anchor regimen across all age groups, and especially in settings where ART prescribing follows a public health approach.^7^^,^^15^

We therefore conducted a six-year longitudinal cohort study among children and adolescents receiving HIV care at a large peri-urban treatment center in Uganda to examine the effectiveness of ABC-, AZT-, and TDF-based ART backbones and identified factors associated with unsuppressed VL. This study provides critical evidence to inform the optimization of pediatric and adolescent ART in this context.

## Methods

### Study design

This retrospective cohort study reviewed medical records of children and adolescents aged 19 years and below who were enrolled in the HIV care and treatment clinic at Mildmay Uganda Hospital (MUgH) between 2018 and 2023.

### Study site and population

The study was conducted at MUgH, a peri-urban facility in Wakiso District with a 50-bed capacity. MUgH provides integrated TB–HIV care and antiretroviral therapy to approximately 15,000 active PLHIV.^16^ The study population comprised all children and adolescents aged 19 years and below who were registered and actively followed in the HIV care and treatment clinic between 2018 and 2023. 2018 was the entry point into the study cohort.

### Data collection and management

Data collection and participant recruitment were conducted over a four-month period (September to December 2024). Data on participants' socio-demographic characteristics and medical history were extracted from registers, patient cards, and Electronic Medical Records (EMR). Data entry was performed using Microsoft Excel, with regular accuracy checks against hard copy records to ensure data integrity. Exclusion criteria included participants with missing viral load data across the six-year period (fewer than 12 measurements) and those enrolled into HIV care after 2018. This approach aligned with Ministry of Health guidelines recommending biannual viral load testing for children and adolescents. The low proportion excluded due to missing viral loads (112 of 1145; 9.78%) and the routine nature of programmatic viral load monitoring make it unlikely that these exclusions materially affected internal validity or statistical power.

A flowchart below illustrates the data collection process.

**Figure:**
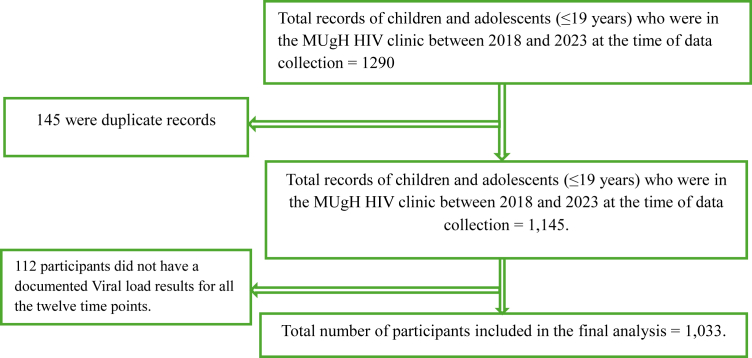

### Statistical analysis plan

We conducted a longitudinal analysis to assess the ART backbone effectiveness among children and adolescents on ART. Descriptive statistics were used to summarize participant characteristics as frequencies and percentages. Overall viral load suppression was used as the primary marker of ART effectiveness across the follow up period. Viral load was measured in copies/mL, and suppression initially categorized as suppressed (≤200), low-level viremia (201–999), and high-level viremia (≥1000). Viral load suppression (VLS) status by ART backbone at the final 2023 measurement was compared using a chi-square test or fishers exact as appropriate and was subsequently reclassified into two categories; suppressed (≤200) and unsuppressed (≥201) for analysis of associated factors. Mixed-effects logistic regression models with participant-specific random intercepts and time slopes, assuming an unstructured covariance, were fitted to account for within-participant correlation arising from repeated measures. The primary outcome was VLS, defined as ≤200 copies/mL and treated as a binary variable (suppressed vs. unsuppressed). The primary predictor was the ART backbone regimen (ABC-, AZT-, or TDF-based). Viral load was modeled both as a continuous (log_10_-transformed) and as categorical variable at 12 time points (2018–2023) for visualization purposes. Mixed-effects logistic regression models assumed a binomial distribution with a logit link, conditional independence of repeated viral load measurements given participant-level random effects, and normally distributed random intercepts and slopes for time. Continuous covariates, including age, were assumed to have a linear relationship with the log-odds of viral suppression. Missing data was assumed to be missing at random. Model development followed three steps: (1) univariable screening (p < 0.20); (2) multivariable modeling adjusting for age (centered), sex, caregiver type, WHO stage, regimen change, number of ART changes, and age at ART initiation; and (3) Third, model refinement included testing interaction terms between ART regimen and time; these were not retained in the final model because they did not improve model fit. This step also involved comparison of competing models. Model 2 log likelihood minus 2988.676, Wald 77.17, AIC 6009.35. Model 3 minus 2997.983, Wald 59.94, AIC 6025.97. Model 4–2988.262, Wald 77.99, AIC 6010.52. Model 5 minus 2986.1803, Wald 82.29, AIC 6004.36, selected final. Age was retained as a continuous variable and number of ART changes as categorical based on improved fit and interpretability. Model level Wald tests were used during refinement rather than individual ordinal Wald statistics in the final tables. Marginal plots were used to estimate and visualize predicted mean log10 viral load and VL suppression probabilities, with time categorized into twelve equally spaced biannual viral load measurement periods across the six year follow up. All analyses were conducted in Stata 17.0 at p < 0.05.

### Ethical considerations

Ethical approval was obtained from the Mildmay Uganda Research Ethics Committee (MUREC) (#REC REF 0201-2024) and then Uganda National Council for Science and Technology (HS3873ES). A waiver of consent was granted by MUREC for using de-identified retrospective data.

### Role of the funding source

The funder of the study had no role in study design, data collection, data analysis, data interpretation, writing of the report, or the decision to submit this manuscript for publication.

## Results

Among 1033 participants, 592 (57.3%) received TDF-based regimens, 338 (32.7%) ABC-based, and 103 (10.0%) AZT-based. Age distribution was: 271 (80.2%) on ABC were aged 5–14 years, while 524 (88.5%) on TDF were older than 14 years; none under five years were on TDF or AZT. Females comprised 162 (47.9%) of ABC users, 49 (47.6%) of AZT, and 334 (56.4%) of TDF. A documented caregiver was noted for 190 (56.2%) on ABC, 53 (51.5%) on AZT, and 281 (47.5%) on TDF; most caregivers were parents (82.5%, 85.4%, and 81.6% respectively). Education records showed primary level in 78 (23.1%) ABC, 29 (28.2%) AZT, and 179 (30.2%) TDF users, while secondary education was highest in TDF (154, 26.0%). Most participants resided in Kampala Metropolitan Area (over 94% in each group). The majority had been on ART for over five years (56.2% ABC, 82.5% AZT, 86.8% TDF), and most were in WHO stage 1 (93.2%, 98.1%, 98.8%) as shown in Table 1 below.

**Table 1:** Social demographic and clinical characteristics of study participants by ART regimens.

Variable	Categories	ABC based (N = 338 32.72%)	AZT based (N = 103 9.97%)	TDF based (N = 592 57.31%)
Age	Mean (SD)	11 (3.8)	13 (3.3)	17 (1.8)
Age	0–9	126 (37.3)	12 (11.7)	0 (0.0)
	10–14	173 (51.2)	55 (53.4)	68 (11.5)
	>15	39 (11.5)	36 (34.9)	524 (88.5)
Sex	Female	162 (47.9)	49 (47.6)	334 (56.4)
	Male	176 (52.1)	54 (52.4)	258 (43.6)
Caretaker type	Parent	279 (82.5)	88 (85.4)	483 (81.6)
	Grand parent	21 (6.2)	6 (5.8)	26 (4.4)
	Sibling	6 (1.8)	2 (1.9)	32 (5.4)
	Others	32 (9.5)	7 (6.8)	51 (8.6)
Education level	Primary	78 (23.1)	29 (28.2)	179 (30.2)
	Secondary	14 (4.1)	11 (10.7)	154 (26.0)
	Tertiary	2 (0.6)	0 (0.0)	4 (0.7)
	Not documented	244 (72.2)	63 (61.2)	255 (43.1)
Residence	Kampala Metropolitan Area (KMA)	320 (94.7)	99 (96.1)	557 (94.1)
	Outside KMA	18 (5.3)	4 (3.9)	35 (5.9)
Participant Age at the start of ART (Years)	<1	82 (24.3)	25 (24.3)	50 (8.5)
	1–2	87 (25.7)	24 (23.3)	115 (19.4)
	>2–5	119 (35.2)	30 (29.1)	195 (32.9)
	6–12	47 (13.9)	22 (21.4)	186 (31.4)
	>12	3 (0.9)	2 (1.9)	46 (7.8)
ART initiation at MUgH	Yes	320 (94.7)	98 (95.1)	565 (95.4)
	No	18 (5.3)	5 (4.9)	27 (4.6)
Regimen change	No	37 (10.9)	0 (0.0)	42 (7.1)
	Yes	301 (89.1)	103 (100.0)	550 (92.9)
Number of Regimen change the child ever had	None	37 (11.0)	0 (0.0)	42 (7.1)
	Once	261 (77.2)	27 (26.2)	428 (72.3)
	≥Twice	40 (11.8)	76 (73.8)	122 (20.6)
Duration on ART (years)	<1	23 (6.8)	0 (0.0)	17 (2.9)
	1–2	36 (10.7)	0 (0.0)	18 (3.0)
	>2–5	89 (26.3)	18 (17.5)	43 (7.3)
	>5	190 (56.2)	85 (82.5)	514 (86.8)
WHO stage	Stage 1	315 (93.2)	101 (98.1)	585 (98.8)
	Stage 2 & above	23 (6.8)	2 (1.9)	7 (1.2)

ABC, abacavir; AZT, zidovudine; TDF, tenofovir disoproxil fumarate; MUgH, Mildmay Uganda Hospital; KMA, Kampala Metropolitan Area; ART, antiretroviral therapy; WHO, World Health Organization.

### Effectiveness by ART backbone at final 2023 measurement

Table 2 shows that overall, 863 of 1033 participants (83.5%) achieved viral suppression. The highest viral suppression was observed in the TDF group with 502 of 592 participants (84.8%), followed by 280 of 338 (82.8%) on ABC, while the lowest suppression occurred in the AZT group with 81 of 103 (78.6%). Low-level viremia was infrequent, highest in the TDF group (17 of 592, 2.9%) and lowest in the AZT group (2 of 103, 1.9%), with 7 of 338 (2.1%) on ABC. High-level viremia was observed in 144 participants (13.9%) overall, highest in the AZT group (20 of 103, 19.4%) and lowest in the TDF group (73 of 592, 12.3%), while 51 of 338 (15.1%) were on ABC. Viral load outcomes were comparable across regimens (p = 0.320).

**Table 2:** Viral load suppression status by antiretroviral therapy (ART) backbone at final 2023 measurement.

VL Suppression category		ABC based (n = 338)	AZT based (n = 103)	TDF based (n = 592)	Total (n = 1033)	p value
Classified by level of viremia	Suppressed	280 (82.8%)	81 (78.6%)	502 (84.8%)	863 (83.5%)	0.320 (Fishers' exact)
	Low-level viremia	7 (2.1%)	2 (1.9%)	17 (2.9%)	26 (2.5%)	
	High-level viremia	51 (15.1%)	20 (19.4%)	73 (12.3%)	144 (13.9%)	
Classified by suppression status	Suppressed	280 (82.8%)	81 (78.6%)	502 (84.8%)	863 (83.5%)	0.273 (Chi2)
	Non Suppressed	58 (17.2%)	22 (21.7%)	90 (15.2%)	170 (16.5%)	

ABC, abacavir; AZT, zidovudine; TDF, tenofovir disoproxil fumarate; VL, viral load.

### Longitudinal analysis of viral suppression over six years

**Fig. 1:**
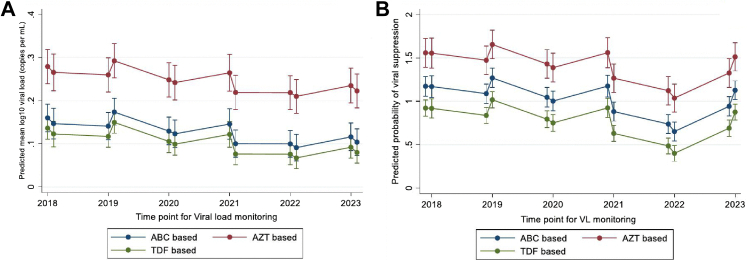
**Predictions of Log-Transformed Viral Load and Viral Suppression by antiretroviral therapy backbone regimen and time point**. Panel A presents predicted mean log10 viral load (Continous) derived from a mixed effects linear regression model with random intercepts for participants. Panel B presents predicted probability of viral suppression derived from a mixed effects logistic regression model using a binary outcome. ART regimen was included as a fixed effect to illustrate overall temporal trends. All error bars in Panels A and B represent 95% confidence intervals of the model based predicted estimates. Abbreviations: ABC, abacavir; AZT, zidovudine; TDF, tenofovir disoproxil fumarate; VL, viral load.

The Fig. 1 below illustrates trends in viral load suppression across twelve measurement points taken twice yearly over the six-year follow-up. TDF-based regimens were associated with the highest overall levels of viral suppression across the study period, followed by ABC-based regimens, with AZT-based regimens showing the lowest suppression rates. This pattern was consistent when viral load was modeled as a continuous variable and when categorized into binary outcomes (suppressed and unsuppressed), with suppression defined as ≤200 copies/mL. Across all time points, participants on TDF maintained superior viral control, while those on ABC showed moderate performance and those on AZT lagged behind.

### Bivariable and multivariable logistic regression analysis of factors associated with viral load non-suppression across all time points

On Bi variable analysis, compared with children on ABC-based regimens, those on AZT-based regimens had significantly higher odds of virological non-suppression (OR 2.84; 95% CI 1.84–4.40; p < 0.001). In contrast, TDF-based regimens were associated with lower odds of non-suppression (OR 0.71; 95% CI 0.53–0.95; p = 0.023). Increasing age was associated with a lower likelihood of non-suppression (OR 0.94 per year; 95% CI 0.91–0.97; p < 0.001). Male sex was associated with higher odds of non-suppression compared with female sex (OR 1.41; 95% CI 1.07–1.84; p = 0.013). Participants cared for by other guardians demonstrated a lower likelihood of non-suppression that was borderline significant (OR 0.57; 95% CI 0.33–1.00; p = 0.049). Time point of viral load testing also showed a significant association, with odds of non-suppression declining over time (OR 0.93 per interval; 95% CI 0.91–0.95; p < 0.001).

In the multivariable model, AZT-based regimens remained significantly associated with higher odds of non-suppression compared with ABC (aOR 2.02; 95% CI 1.20–3.40; p = 0.008). The effect observed for TDF-based regimens was attenuated and no longer significant after adjustment (aOR 1.08; 95% CI 0.68–1.71; p = 0.748). Older age continued to be protective, with each additional year reducing the odds of non-suppression (aOR 0.92; 95% CI 0.86–0.98; p = 0.006). Male sex remained a significant predictor of non-suppression (aOR 1.36; 95% CI 1.03–1.81; p = 0.033). Additionally, later time points of viral load testing were associated with improved suppression (aOR 0.95; 95% CI 0.91–1.00; p = 0.037) as shown below in Table 3.

**Table 3:** Bi variable and multivariable analysis of factors associated with viral load non-suppression across all time points.

Variable	Categories	OR (95% CI)	p value	aOR (95% CI)	p value
Backbone	ABCbased	Ref			
	AZT based	2.84 (1.84–4.40)	<0.001	2.02 (1.20–3.40)	0.008
	TDFbased	0.71 (0.53–0.95)	0.023	1.08 (0.68–1.71)	0.748
Age		0.94 (0.91–0.97)	<0.001	0.92 (0.86–0.98)	0.006
Sex	Female	Ref			
	Male	1.41 (1.07–1.184)	0.013	1.36 (1.03–1.81)	0.033
Documented Caretaker	Yes	Ref			
	No	0.96 (0.73–1.26)	0.766		
Caretaker type	Parent	Ref			
	Grand parent	1.09 (0.59–2.02)	0.778	1.03 (0.54–1.99)	0.919
	Sibling	0.53 (0.22–1.26)	0.149	0.62 (0.25–1.54)	0.307
	Others	0.57 (0.33–1.00)	0.049	0.58 (0.32–1.03)	0.064
Education level	Primary	Ref			
	Secondary	1.10 (0.73–1.67)	0.649	0.66 (0.41–1.05)	0.076
	Tertiary	0.27 (0.03–2.59)	0.255	1.02 (0.67–1.57)	0.914
	Not documented	0.97 (0.70–1.33)	0.840		
Residence	Kampala Metropolitan Area (KMA)	Ref			
	Outside KMA	1.12 (0.63–2.00)	0.695		
Participant Age at the start of ART (Years)	<1	Ref			
	1–2	0.56 (0.36–0.88)	0.012	0.72 (0.45–1.15)	0.164
	>2–5	0.82 (0.55–1.23)	0.338	1.12 (0.72–1.74)	0.609
	6–12	0.78 (0.51–1.20)	0.266	1.37 (0.84–2.25)	0.210
	>12	0.46 (0.18–1.18)	0.104	0.63 (0.17–2.30)	0.488
ART initiation at MUgH	Yes	Ref			
	No	0.77 (0.37–1.62)	0.497		
Regimen change	Yes	Ref			
	No	0.63 (0.32–1.25)	0.188	0.34 (0.06–1.84)	0.211
Number of Regimen change	None	Ref		Reference	–
	Once	0.50 (0.24–1.05)	0.067	0.95 (0.15–6.13)	0.956
	≥Twice	1.47 (0.68–3.17)	0.328	2.52 (0.37–17.03)	0.345
Duration on ART (years)	<1	Ref			
	1–2	0.94 (0.12–7.62)	0.953		
	>2–5	1.56 (0.21–11.50)	0.661		
	>5	0.91 (0.13–6.51)	0.923		
WHO stage	Stage 1	Ref		Reference	–
	Stage 2& above	2.46 (0.95–6.35)	0.063	1.69 (0.57–5.03)	0.349
Time point of VL testing	2018–2023	0.93 (0.91–0.95)	<0.001	0.95 (0.91–1.00)	0.037

ABC, abacavir; AZT, zidovudine; TDF, tenofovir disoproxil fumarate; MUgH, Mildmay Uganda Hospital; KMA, Kampala Metropolitan Area; ART, antiretroviral therapy; WHO, World Health Organization; VL, viral load.

## Discussion

This six-year longitudinal cohort study among children and adolescents receiving HIV care at a large peri-urban HIV treatment center in Uganda provides important real-world evidence on the performance of different ART backbones in achieving VL suppression. We observed that overall VLS was high, exceeding 80% across the cohort, but still below the Joint United Nations Programme on HIV and AIDS (UNAIDS) 95% suppression target, and notable differences were evident between the various backbone regimens. TDF regimens achieved highest suppression (84.8%), ABC followed (82.8%), while AZT (78.6%) remained lowest.

The superior performance of TDF-based regimens observed in this study aligns with the broader evidence base supporting TDF as a potent, well-tolerated component of first-line ART.^6^^,^^8^ TDF's pharmacologic profile, once-daily dosing, and widespread availability through global initiatives such as PEPFAR and the Global Fund^17^ may contribute to improved adherence and consistent virological outcomes.^18^, ^19^, ^20^ The majority of TDF recipients in our cohort were older than 14 years, an age group more likely to benefit from better adherence, supported by a developing sense of personal responsibility and tailored support systems.^21^ Conversely, ABC was more commonly used among younger children, in whom challenges such as liquid formulations, weight-based dosing, and caregiver dependence may have moderated virological outcomes.^22^

In contrast, AZT-based regimens demonstrated both the lowest suppression (78.6%) and the highest rates of high-level viremia (19.4%). Even after adjusting for age, sex, and other confounders, AZT use was associated with two-fold higher odds of non-suppression compared with ABC. Several factors may underlie this observation. AZT is associated with more frequent hematologic toxicity and gastrointestinal side effects, which may compromise adherence.^23^^,^^24^ Furthermore, programmatically, children placed on AZT backbones may have been transitioned due to prior regimen, failure, toxicity, intolerance which might introducing a selection bias toward those with more complex clinical histories. Importantly, AZT is twice dosing drugs which complicates and already existing adherence problem in this population this could also explain the results.

Age emerged as a significant predictor of VL outcomes in both bi variable and multivariable models. With each additional year of age, the odds of non-suppression decreased by 8 percent (aOR 0.92; 95% CI 0.86–0.98). This finding is consistent with prior studies showing that younger children, particularly those under five years, are more vulnerable to suboptimal virological responses due to factors such as complex dosing regimens, palatability issues with pediatric formulations, and reliance on caregivers for medication administration.^25^ Furthermore, immunologic immaturity in early life and higher baseline viral set points may contribute to delayed suppression in younger populations. Adolescents, despite facing unique adherence challenges related to stigma, disclosure, and psychosocial stressors, have also shown improvements in suppression rates in recent years,^13^ likely reflecting enhanced adolescent-friendly services, dedicated peer support programs, and national guideline shifts prioritizing potent regimens like dolutegravir (DTG).^6^^,^^26^ The strong age gradient observed in our study emphasizes the need for age-specific interventions, including child-friendly formulations, caregiver counseling, and adolescent-tailored adherence support.^25^^,^^27^

We also found that male participants were significantly more likely to experience non-suppression compared with females (aOR 1.36; 95% CI 1.03–1.81). While sex differences in pediatric HIV outcomes are not consistently reported, some studies have suggested that girls often demonstrate better clinic engagement and adherence, possibly reflecting sociocultural dynamics within households where female children may receive closer supervision or care.^28^ Alternatively, biological factors such as pharmacokinetic variations or differences in immune activation between sexes might play a role, though evidence in pediatric populations remains limited.^29^ Regardless, these findings suggest that male children and adolescents may require additional adherence counseling and closer monitoring.

An additional important finding was the effect of the time point of VL testing on suppression outcomes. Each advancing year between 2018 and 2023 was associated with improved odds of suppression in both bi variable and multivariable analyses. This suggests a temporal improvement in treatment success over the study period. The improvement likely reflects programmatic gains over time, including wider availability of effective ART regimens, enhanced clinic support systems, and strengthening of national monitoring and supply chains.^30^ It may also reflect growing experience among healthcare teams and improved caregiver and patient education. These findings emphazise the dynamic nature of ART outcomes and the value of continuous quality improvement within national HIV programs.

Our findings support prioritizing tenofovir-based regimens in resource-limited settings, given higher suppression rates. AZT outcomes remain poorer, reinforcing policy shifts away from its routine use. Programs should ensure child-friendly formulations, such as tenofovir alafenamide (TAF), provide age- and sex-sensitive adherence support, and individualize regimen choice based on clinical, immunologic, and social factors rather than solely on age or weight.

This study was conducted under a public health approach to ART delivery, where simplified and standardized regimens are implemented across wide age groups, and dolutegravir (DTG) is now the preferred anchor regimen. Optimizing the NRTI backbone in such settings is crucial to maximize DTG's high barrier to resistance and progress toward the UNAIDS 95-95-95 targets. Understanding how different backbones perform in routine care informs not only regimen selection but also supply chain management, pediatric formulation planning, and the design of differentiated service delivery models.

The study has several strengths and limitations. Its strengths include a large cohort and six year follow up period, which enabled a robust assessment of viral suppression trends. The analysis was strengthened by both bivariable and multivariable modeling that adjusted for key confounders. In addition, the use of routine programmatic data enhances the relevance of the findings to real world clinical practice. Residual confounding from unmeasured factors such as adherence or socioeconomic status is possible, and regimen allocation was not randomized. Although a small number of participants were excluded (112, 9.87%), these individuals may have been lost to follow-up or died, and those who became non-suppressed and subsequently died are not represented. This warrants cautious interpretation of the findings, as their exclusion introduces potential selection bias. However, the substantial sample remaining at the final time point likely provides meaningful representation (170, 16.4%) of non-suppressing children and adolescents. Data were collected from a single peri-urban study site, which may limit generalizability. Finally, while VL suppression is a critical marker of treatment success, we did not evaluate longer-term outcomes such as resistance profiles, immunologic recovery, or clinical events.

TDF backbones achieved the highest viral suppression, ABC was second, and AZT performed poorest, emphasizing the need for age- and sex-specific support to improve outcomes in children and adolescents living with HIV. Future prospective studies incorporating adherence metrics, resistance testing, and patient-reported outcomes are needed to clarify mechanisms behind backbone differences and evaluate other tenofovir-based regimens in this population. With widespread DTG adoption, ongoing assessment of backbone optimization remains essential to ensure effective, context sensitive, and sustainable antiretroviral therapy in resource-limited settings.

## Contributors

CA, BK, JE, SN, and JN conceived and designed the study. CA acquired funding. CA and JE had full access to the raw data, accessed and verified the underlying data, and take responsibility for the integrity and accuracy of the data analysis. CA conducted the statistical analysis. CA, BK, CS, JE, SN, IM, and JN contributed to data collection and interpretation. All authors contributed to drafting or critically revising the manuscript, approved the final version, and accept responsibility for the decision to submit for publication.

## Data sharing statement

The de identified dataset underlying this study is available from the corresponding author and JE (jd.emunyu@gmail.com) upon reasonable request, subject to institutional data sharing policies and ethical approval.

## Declaration of interests

We declare no competing interests.
